# Thyroid cancers of follicular origin in a genomic light: in-depth overview of common and unique molecular marker candidates

**DOI:** 10.1186/s12943-018-0866-1

**Published:** 2018-08-08

**Authors:** Natalia Pstrąg, Katarzyna Ziemnicka, Hans Bluyssen, Joanna Wesoły

**Affiliations:** 1 Laboratory of High Throughput Technologies, Institute of Molecular Biology and Biotechnology, Faculty of Biology, Adam Mickiewicz University in Poznan, ul Umultowska 89, 61-614 Poznań, Poland; 2 Department of Endocrinology, Metabolism and Internal Diseases, Poznan University of Medical Sciences, Poznan, Poland; 3 Department of Human Molecular Genetics, Institute of Molecular Biology and Biotechnology, Faculty of Biology, Adam Mickiewicz University in Poznan, ul Umultowska 89, 61-614 Poznań, Poland

**Keywords:** Thyroid cancer, Biomarkers, NGS, Molecular markers, PTC, FTC, ATC

## Abstract

In recent years, thyroid malignances have become more prevalent, especially among women. The most common sporadic types of thyroid tumors of follicular origin include papillary, follicular and anaplastic thyroid carcinomas. Although modern diagnosis methods enable the identification of tumors of small diameter, tumor subtype differentiation, which is imperative for the correct choice of treatment, is still troublesome. This review discusses the recent advances in the field of molecular marker identification via next-generation sequencing and microarrays. The potential use of these biomarkers to distinguish among the most commonly occurring sporadic thyroid cancers is presented and compared. Geographical heterogeneity might be a differentiator, although not necessarily a limiting factor, in biomarker selection. The available data advocate for a subset of mutations common for the three subtypes as well as mutations that are unique for a particular tumor subtype. Tumor heterogeneity, a known issue occurring within solid malignancies, is also discussed where applicable. Public databases with datasets derived from high-throughput experiments are a valuable source of information that aid biomarker research in general, including the identification of molecular hallmarks of thyroid cancer.

## Background

Thyroid cancer (TC) is one of the most frequent endocrine malignancies, accounting for 3–4% of cancers [1], and its occurrence has increased by approximately 5% on a yearly basis, with higher prevalence in females than in males (20.6 vs. 6.9 new cases per 1000 persons) [2]. The number of newly diagnosed cases has risen dramatically in the last 10 years, which could be partially ascribed to the availability of more sensitive diagnostic tools, i.e., ultrasonography and fine-needle aspiration (FNA) and the smaller size of diagnosed tumors. However, over diagnosis is also an issue because its occurrence rate has risen 15-fold since 2003, whereas mortality rates have not changed [3].

In general, the 5- and 10-year survival rates for TC patients are excellent (approx. 98%) but are related to the age of the patient at the time of diagnosis and the cancer subtype [1, 4, 5].

**Fig. 1:**
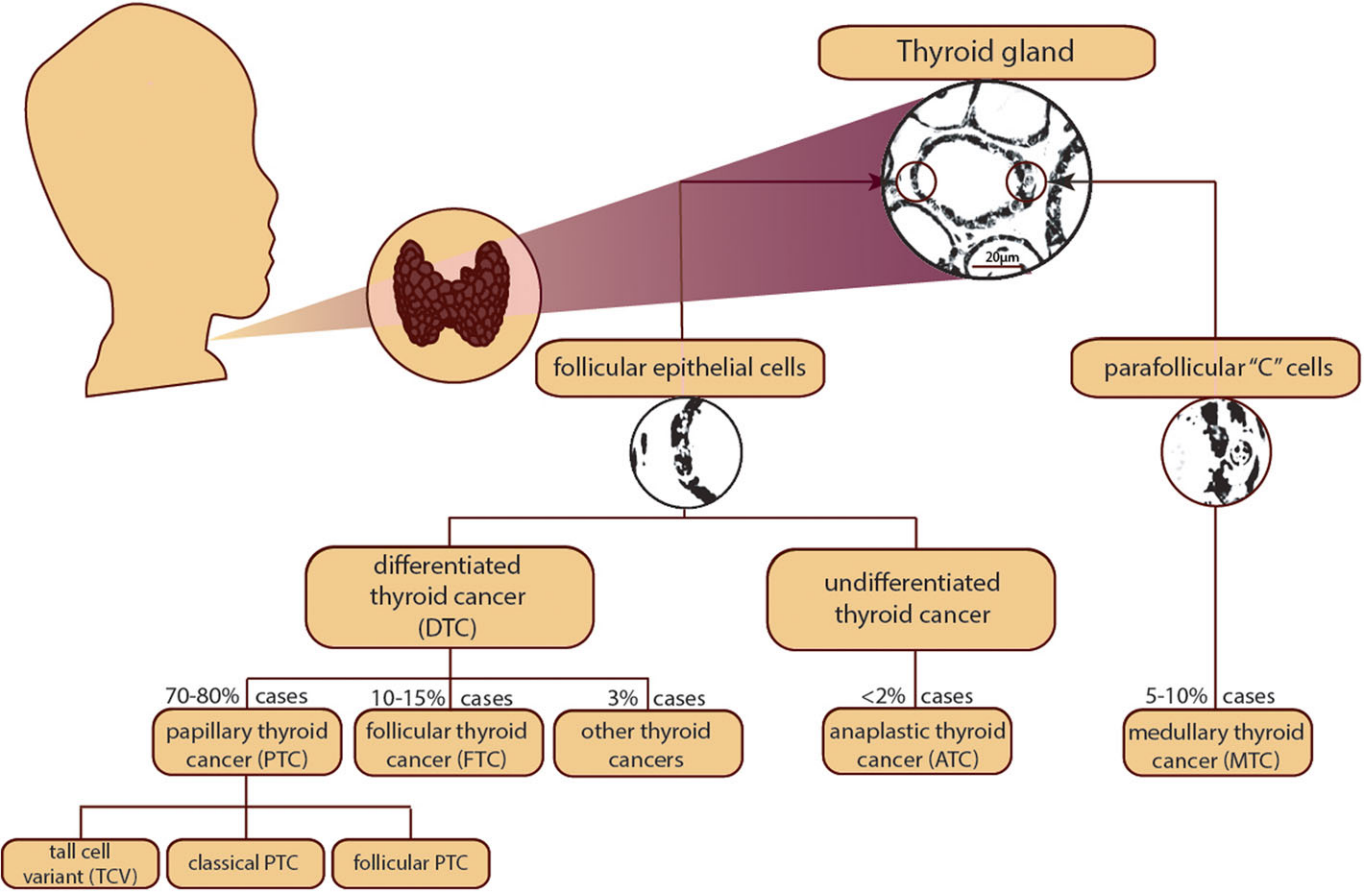
Overview of thyroid cancer types and their origins

Both papillary (PTC) and follicular thyroid carcinoma (FTC) arise from follicular epithelial thyroid cells involved in iodine metabolism. PTC and FTC, together with the less common Hürtle cell carcinoma, are classified as differentiated thyroid cancer (DTC, see Fig. 1) [6, 7]. Both PTC and FTC progress slowly and are generally characterized by good prognosis, especially if diagnosed early [5].

Undifferentiated anaplastic thyroid carcinoma (ATC) is the most aggressive TC type. Although ATC also originates from follicular cells, similar to PTC and FTC, it does not possess their original biological properties [8]. ATC represents 2–5% of cases, (77% in women) with the worst prognosis and a 5-year survival rate of 5% [3]. ATC is insensitive to conventional methods of treatment [9].

In contrast, medullary thyroid cancer (MTC) is derived from parafollicular thyroid “C” cells, which produce calcitonin [2].

The majority of TC cases are sporadic, with only 5% of DTC characterized as familial (mostly PTC) and ~ 25% of MTC inherited as an autosomal trait [10]. Only sporadic tumors are analyzed in this review.

Although most mutations found in TC differ among types, certain DNA alterations were found to be common in more than one subtype. As discussed later in this review, ATC tumors appear to derive from other differentiated tumors and thus possess a large overlap with mutations present in DTCs, such as *TMPRSS4*. Mutations in certain genes, e.g., *CHEK2,* are reported in both PTC and FTC, although not with the same prevalence [11, 12], and their potential contribution to TC carcinogenesis is described in the respective paragraphs. In this work, we focus on tumor heterogeneity and the mutation burden carried by thyroid tumors, as tested primarily by high-throughput methods performed within larger genomic projects, including The Cancer Genome Atlas (TCGA).

We gathered the data from RNA expression and DNA sequencing experiments and identified potential genetic biomarkers of disease progression. Genome-wide association studies (GWAS) as well as sequencing and microarrays were considered. In this work, we present an overview of the available biomarkers candidates for progression and development of thyroid cancer and drivers of carcinogenesis, as discussed in detail in the respective sections. All gene functions were inferred using GeneCards (www.genecards.org) [13].

Genome-wide studies significantly aid in the identification of cancer-specific germline and somatic mutations, which can contribute to more sensitive diversification of cancer subtypes and facilitate early diagnosis. Identification of disease-specific point mutations can accelerate the evaluation of candidate target genes for therapeutic drugs and the search for novel driver mutations. However, the identification of polymorphisms (SNPs) could additionally improve prognosis and patient outcomes.

### Common genetic determinants of thyroid cancer subtypes

In recent years, the development of sequencing and microarray technologies has permitted a whole-genome search for TC-linked or associated genes. Genome-wide association studies (GWAS) are a highly potent method for identification of high-incidence single nucleotide polymorphisms (SNPs) and copy number variations (CNVs). Recently, GWAS were used to study large TC patient cohorts [14–17] and were followed by studies confirming the findings [18–27]. Mutation hot spots identified through GWAS (microarray, next-generation sequencing (NGS) and high-resolution melting (HRM)) are collected in Table 1. Specific SNPs could be associated with susceptibility to DTC (mostly papillary and follicular) in single or multiple populations with variable strength.

**Table 1:** Somatic mutations associated with susceptibility to differentiated thyroid cancers

Chromosomal location	DbSNP identification No.	Gene	Gene function	Cancer type	Tested population	Literature
1p31.3	rs334725	*NFIA*	Nuclear transcription factor	PTC, FTC	Icelandic, American, Dutch, Spanish	Gudmundsson et al.*,* 2012
1q42.2	rs12129938	*PCNXL2*	Correlated with tumorigenesis of colorectal carcinomas	DTC	Icelandic, American, Spanish, Dutch	Gudmundsson et al.*,* 2017
2q35	rs966423, rs6759952	*DIRC3*	lincRNA	PTC, FTC	Icelandic, American, Dutch, Spanish, Polish	Gudmundsson et al.*,* 2012, Liyanarachchi et al.*,* 2013
				DTC	Italian, Polish, Spanish, English	Köhler et al.*,* 2013
3q25.32	rs7617304	*RARRES1*	Membrane protein gene responsive to retinoid acid	DTC	Italian	Köhler et al.*,* 2013
3q26.2	rs6793295	*LRCC34* near *TERC* (missense)	RNA telomerase	PTC, FTC	Icelandic, American, Spanish, Dutch	Gudmundsson et al.*,* 2017
4q34.3	rs17739370 TT variant	*NEIL3*	DNA repair, BER	DTC	Italian	Cipollini et al.*,* 2016
5q22.1	rs73227498	*NREP* and *EPB41L4A*	Intergenic region	PTC, FTC	Icelandic, American, Spanish, Dutch	Gudmundsson et al.*,* 2017
5	rs13184587	*ARSB* intron	Intron of lysosomal sulfatase	DTC	Italian	Figlioli et al.*,* 2014
7q21	rs10238549, rs7800391	*IMMP2L*	Processing of signal peptides in mitochondrial membrane	DTC	Italian	Köhler et al.*,* 2013
8p12	rs2439302	*NRG1*	membrane glycoprotein, signaling mediator	PTC, FTC	Icelandic, American, Dutch, Spanish	Gudmundsson et al.*,* 2012
8q24	rs6983267	ncRNA	N/A	PTC	English	Jones et al.*,* 2012
9q3.3	rs10781500	*SNAPC4*	Large subunit of the DNAP complex	DTC	Italian	Köhler et al.*,* 2013
9q22.33	rs965513, rs1867277 (5’UTR region), rs71369530	Proximity to *FOXE1*	Deregulation of thyroid morphogenesis	PTC	Icelandic, Caucasian, Asian, Cuban, English, Belarussian, French Polynesian	Jones et al.*,* 2012, Gudmundsson et al.*,* 2009, Liyanarachchi et al.*,* 2013, Damiola et al.*,* 2014, Wang et al.*,* 2016, Pereda et al.*,* 2015, Maillard et al.*,* 2015
10q24.33	rs7902587	near *OBFC1*	Stimulator of DNA replication initiation factor	PTC, FTC	Icelandic, American, Spanish, Dutch	Gudmundsson et al.*,* 2017
11	rs1801516	*ATM*	Cell-cycle checkpoint, response to DNA damage	DTC	Cuban women after multiple pregnancies, French Polynesian	Pereda et al.*,* 2015, Maillard et al.*,* 2015
13	rs1220597	*SPATA13* intron	Regulation of cell migration and adhesion, guanine nucleotide exchange factor	DTC	Italian	Figlioli et al.*,* 2014
14q13.3	rs116909374	*NKX2–1*	Thyroid-specific transcription factor	PTC, FTC	Icelandic, American, Dutch, Spanish, Polish	Gudmundsson et al.*,* 2012, Liyanarachchi et al.*,* 2013
14q13.3	rs944289	Close to *NKX2–1*	Thyroid-specific transcription factor	PTC, FTC	Icelandic, Cuban, English, American, Polish, French Polynesian	Jones et al.*,* 2012, Gudmundsson et al.*,* 2009, Liyanarachchi et al.*,* 2013, Pereda et al.*,* 2015, Maillard et al.*,* 2015
14	241(Thr > Met)	*XRCC3*	DNA repair, homologous recombination	DTC	Chinese, Iranian, Caucasian Portuguese	Wang et al.*,* 2015, Fayaz et al.*,* 2014, Bastos et al.*,* 2009
14	rs10136427	*BATF*	Transcription factor, negative regulator of AP-1/ATF transcriptional events	DTC	Italian, Polish, Spanish	Figlioli et al.*,* 2014
15q22.33	rs2289261, rs56062135	*SMAD3*	Transcriptional modulator	PTC, FTC	Icelandic, American, Spanish, Dutch	Gudmundsson et al.*,* 2017
20	rs7267944	*DHX35*	RNA helicases	DTC	Italian, Polish, Spanish	Figlioli et al.*,* 2014

Variants determined by GWAS. *DTC* Unspecified differentiated thyroid cancer, *PTC* Papillary thyroid cancer, *FTC* Follicular thyroid cancer

Sixteen case/control studies allowed identification of 27 SNPs located primarily within the coding regions (see Table 1). Only rs6983267 was located in the non-coding RNA; rs1220597, rs73227498, and rs13184587 were located in the introns; and rs965513, rs1867277, rs71369530 and rs944289 were located in proximity to *NKX2–1*. This observation might stem from the fact that most microarray and NGS experiments are focused on transcriptome analysis and can be biased against regulatory or non-coding fragments. TC-associated genes are often connected to DNA-damage repair or transcription.

Using a slightly different approach, Gudmundsson et al.*,* selected 22 SNPs based on a score of high association with high levels of thyroid stimulating hormone in a GWAS study of over 27,000 samples from an Icelandic population [16]. The results of genotyping of 561 samples of the non-medullary type were compared with over 40,000 controls from different populations (Dutch, American and Spanish). Three variants proved to be significantly correlated, namely, rs966423 in non-coding RNA-*DIRC3* (OR = 1.34, *P* = 1.3·10^− 9^), rs2439302 membrane glycoprotein involved in cell signaling *NRG1* (OR = 1.36, *P* = 2.0·10^− 9^) and rs116909374 in thyroid-specific transcription factor *NKX2–1* (OR = 2.09, *P* = 4.6·10^− 11^), and their functions in thyroid tumorigenesis are still unknown.

The ThyroSeq microarray panel (ThyroSeq) is widely used and offers the possibility of testing more than 1000 hotspots in 14 TC-related genes and over 40 fusions simultaneously. Nikiforova and Nikiforov tested over 800 TC samples of all types using ThyroSeq panels, thus proving its usefulness in detection and classification of cancerous tissue [28–30].

Figlioli et al.*,* performed SNP genotyping of an Italian population (case/controls: 1437/1534), validated in DTC patients from Poland (case/controls: 448/424) and Spain (case/controls: 375/408) [14]. The strongest correlation among all tested cohorts was found for rs10136427 localized in transcription regulator *BATF*, (OR = 1.40, *P* = 4.35·10^− 7^) and rs7267944 in putative RNA helicase *DHX3* (OR = 1.39, *P* = 2.13·10^− 8^).

Gudmundsson et al.*,* published a follow-up study in Icelandic, Dutch, Spanish and 2 American populations (case/controls: 1003/278,991, 85/4956, 83/1612, 1580/1628 and 250/363, respectively) confirming 5 novel loci associated with non-medullary thyroid cancer (P_combined_ < 3 × 10^− 8^), i.e., rs12129938, rs6793295, rs73227498, and two independently associated variants, i.e., rs2289261 (OR = 1.23; *P* = 3.1·10^–9)^ and rs56062135 (OR = 1.24; *P* = 4.9·10^− 9^) [31].

Applying a presumption that the DNA repair genes of base (BER) or nucleotide (NER) excision repair pathways might be involved in TC tumorigenesis, Cipollini et al.*,* genotyped known SNPs in 450 case-control paired DTC samples from an Italian population [32]. The TT variant of base excision repair gene *NEIL3*, which codes a DNA glycosylase, was associated with increased risk of DTC. Another GWAS study on an Italian population performed by Köhler et al. associated mutations in non-coding RNA genes *DIRC3*, *RARRES1*, *SNAPC4* and *IMMP2L* with increased DTC in a high-incidence population of 690 cases and 497 controls and confirmed this finding in 3 low-incidence populations (total of 2958 cases and 3727 controls) [15] (See Table 1). *SNAPC4* encodes a large subunit of the RNA-activation protein complex, and *RARRES1* and *IMMP2L* are transmembrane proteins.

### Papillary thyroid Cancer (PTC)

Derived from follicular cells, papillary thyroid cancer is named after its cyto-architecture and can be further divided into 3 subtypes based on histotype: tall cell variant (TCV), follicular, and classical (most common) [33]. According to TCGA, up to 70% of somatic PTC drivers are found in activators of the MAPK pathway and include *BRAF*, *RAS* and rearrangements of the *RET* and *NTRK1* genes [5] (See Table 2). The alterations are generally thought to be mutually exclusive in PTC [34–37], but contradictory data have emerged [38–41]. Other mutations such as *PTEN* and *PIK3CA* [42] have been reported at lower frequencies (2/86 (2.32%) and 3/86 (3.48%), respectively). The mutation density is relatively low at 0.41 mutations/Mb for PTC and 0.5 mutations/Mb for TCV. PTC is often multifocal, with a main tumor (> 1 cmØ) and several microcarcinomas [43, 44]. Nodules might be positioned unilaterally or bilaterally in the thyroid lobes. Multifocality is a characteristic of up to 40% of all PTC, [45, 46] leading to aggressiveness and resistance to radioiodine treatment [47]. The clonal origin of each singular carcinoma is not necessarily the same because tumors might arise independently through a series of molecular events, such as chromosome X inactivation [43, 48–52], but certain authors suggest clonal homogeneity between the nodules [49, 53–57].

**Table 2:** Somatic mutations characteristic of PTCs

Gene	Localization	Gene function	Mutation	Defect in cancer	Clinical correlation	Literature
Gene						
*BRAF*	7q34	Serine/threonine kinase, response to cell growth factors	B-type Raf Kinase (chr 7) 2 Missense mutation V600E (T < A 1799), rs113488022	Constitutive activation of MAPK pathway	Positive correlation with age, marker of TCV subtype	TCGA, Kimbrell et al.*,* 2015, Lu et al.*,* 2016, Gandolfi et al.*,* 2013, Kim et al.*,* 2006, Guerra et al.*,* 2012, Sun et al.*,* 2016, Gertz et al.*,* 2016, Iyer et al.*,* 2015, Lee et al.*,* 2016
*CHEK2*	22q12.1	Cell cycle checkpoint kinase	IVS2 + 1G > A, 1100delC or del5395, missense mutation I157T	DNA repair mechanism dysfunctions	Positive correlation with cancer aggressiveness	Siolek et al.*,* 2015, Wójcicka et al.*,* 2014, Kaczmarek-Ryś et al.*,* 2015
*DLL4*	15q15.1	Notch signaling mediator	Patient specific mutations	Promotes angiogenesis	Correlated with presence of lymph node metastases	Le Pennec et al.*,* 2015
*EIF1AX*	Xp22.12	Translation initiation factor, transfer of Met-tRNAf	Hotspot at A113_splice site intron 5/exon 6	Potential driver mutation	N/A	TCGA, Forbes 2011, Martin 2013, Karanamurthy 2016
*FOXE1*	9q22.33	Transcription factor	rs965513 AA, AG; rs944289; c.821C > A, p.P54Q; c.943A > C p.K95Q; c.994C > T, p.L112F	Deregulation of thyroid morphogenesis	Thyroid cancer susceptibility marker	Mond et al.*,* 2015, Gudmundsson et al.*,* 2009, Penna-Martinez et al.*,* 2014
*PIK3CA*	3q26.32	PI3K/AKT/mTOR pathway effector	E545A	Mutation of helical domain	N/A	Lee et al.*,* 2016
*PTEN*	10q23.31	PI3K/AKT/mTOR pathway effector	N/A	Produces a truncated protein	N/A	Xing et al.*,* 2013
*RAS*	11p15.5, 1p13.2, 12p12.1	Signal transduction	H-Ras (chr11), N-Ras (chr1), K-Ras (chr12)	Preferential activation of PI3K-AKT pathway	Positive correlation with cancer aggressiveness	Rossi et al.*,* 2015, Gertz et al.*,* 2016, Abubaker et al.*,* 2008, Zou et al.*,* 2014
*TERT* promoter	5p15.33	Telomerase reverse transcriptase	C > T 1295228 and 1,295,250 C > A at 1295250	Gain of immortality	Positive correlation with cancer aggressiveness	Bae et al.*,* 2016, Liu et al.*,* 2014, Liu et al.*,* 2013, Sun et al.*,* 2016
Chromosomal Abberations						
*RET*	10q11.21	Tyrosine kinase transmembrane receptor	Rearrangements: RET/PTC1, RET/PTC2, RET/PTC3, RET/PTC4. RET/PTC5, RET/PTC6, RET/PTC7, RET/PTC8, RET/PTC9, PCM1-RET, EKLS-RET, FKBP-RET, RET-ANK3, TBL1XR1-RET, AKAP13-RET, ERC1-RET, HOOK3-RET, SPECC1L-RET, ACBD5-RET, ΔRFP-RET	Downstream signaling of MAPK and PI3K pathways, evasion of apoptosis	Common in pediatric PTC, common co-occurrence with BRAF mutation	TCGA, Gertz et al.*, 2*016, Rossi et al.*,* 2015, Hamatani et al.*,* 2014, Corvi et al.*,* 2000, Ciampi et al.*,* 2007, Klugbauer et al.*,* 1998, Salassidis et al.*,* 2000, Saenko et al.*,* 2003, Nakata et al.*,* 1999, Hamatani et al.*,* 2014, Bongarzone et al.*,* 1993, Grieco et al.*,* 1990
Abnormal expression						
*ATP5E*	20q13.32	ATPase subunit 5E	Down-regulation	Disruption of ATP synthesis in mitochondria	Potential PTC biomarker	Hurtado-Lopez et al.*,* 2015
*MUC1*	1q22	Proliferation and signaling of epithelial cells	Overexpression	Leads to propagation of tumorigenesis and metastasis	Poor outcome marker	Renaud et al.*,* 2014
*TMPRSS4*	11q23.3	Serine protease	Overexpression	Migration and metastasis of cancer cells	Malignant tumors	Kebebew et al.*,* 2005, Jarzab et al.*,* 2005, Guan et al.*,* 2015
*YY1*	14q32.2	Transcription factor	Overexpression	Leads to increased cell proliferation	Positive correlation with age	Arribas et al.*,* 2015
Regulation Of Expression						
micro RNA	Xp11.3	Regulation of expression of affiliated genes	let-7 miRNA overexpression	Disruption of regulatory pathways (e.g. DNA damage response, stress response), propagation of cancer growth and expansion through down/up-regulation of target genes	N/A	Salajegheh et al.*,* 2016, Yoruker et al.*,* 2016, Lee et al.*,* 2013, Zhang et al.*,* 2010, Lei et al.*,* 2016, Hong et al.*,* 2016, Samsonov et al.*,* 2016, Hu et al.*,* 2017
	9p21.3		miR-31 overexpression			
	8q24.3		miR-146b overexpression			
	19q13.41		miR-151-5p overexpression			
	10q24.32		miR-221 overexpression			
	Xp11.3		miR-222 overexpression			
	17q23.1		miR-21 down-regulation			
	9q34.3		miR-126 down-regulation			
	19p13.12		miR-20b			
	Xq26.2		miR-639			

### Genetic alterations in kinases

#### BRAF

The most common somatic mutation occurring in PTC is a mis-sense *BRAF* mutation resulting in thymine-to-adenine substitution at position 1799 of the B-type Raf Kinase (*BRAF*) gene. This mutation leads to a valine-to-glutamate substitution at codon 600 of the BRAF protein (BRAF^V600E^) and constitutive activation of the MAPK signaling pathway via activation of the G-coupled receptor in the membrane [58–60], and it is common for several cancers, including non-small cell lung cancer and melanomas. [59–61]. BRAF is an activator of BRAF-activated non-coding RNA (*BANCR*), which regulates many cellular processes, including tumorigenesis, metastasis and, apoptosis [62]. BRAF can function as both a tumor suppressor and disease progression factor [63]. BRAF^V600E^ is typical for TCV and classical subtypes, whereas *RAS* mutations predominantly drive the follicular subtype [33, 64]. This dependence, in combination with the various prevalence of driver mutations in populations, might explain certain of the disparities between different studies.

Recently, the potential heterogeneity of *BRAF* mutants (intra- and inter-tumoral) has been emphasized using both traditional methods (PCR verified by Sanger sequencing) as well as novel techniques such as exome capture and pyrosequencing. Kimbrell et al.*,* tested 57 tumors from 27 patients for the presence of the *BRAF* ^*V600E*^ mutation [65]. The results were discordant between primary and secondary tumors in 10 out of 27 cases, but no significant histological changes were observed. However, the irregularity of the tumor edge appears to indicate its metastatic origin. No correlation was detected for the lobe positioning of the concordant and discordant nor the size of *BRAF*-positive and negative tumors. Sun et al.*,* showed (*n* = 455) that 75.5% of the patients in a Chinese population harbored a *BRAF*^*V600E*^ mutation, which was significantly correlated with increasing patient age [66]. In contrast, the rate of *BRAF*^*V600E*^ mutations was two times lower in children than in adults [67]. One of 14 pediatric patient samples was positive for concomitant *BRAF* mutation and *RET/PTC3* rearrangement (see below). Lu et al.*,* identified *BRAF*^*V600E*^ mutation as the most common using deep sequencing of 21 foci from 8 patients [68]. The experiments confirmed that multifocal TC could be heterogeneous and that *BRAF* is not necessarily the driver because up to 75% of the clones had independent clonal origins. Those results were supported by reports from other groups in which foci did not share the same mutation patterns [48, 69–71]. Gandolfi et al.*,* tested 37 primary PTC tumors and 95 metastases in adults and found that 43.9% of the samples were *BRAF*-positive, but no correlation was observed with metastasis. The allele percentage shows that *BRAF* mutations are heterogeneous and rarely a result of a clonal event [69, 72]. De Biase et al.*,* tested the distribution of neoplastic cells in *BRAF*^*V600E*^-positive tumors (*n* = 85/155) [51]. The percentage of cells harboring a mutated *BRAF* allele present in each sample varied from less than 30% (*n* = 9/85) to 80% (*n* = 39/85). Down-regulation of the transcript was observed in paired PTC tumor samples and normal adjacent tissues. Real-time PCR shows that the down-regulation of *BANCR* correlates with patient prognosis with consideration of tumor size, number of nodules, stage, gender, metastasis and extrathyroidal extensions but not with age.

#### PIK3CA

Mutations in *PIK3CA*, a catalytic subunit of the phosphatidylinositol 3-kinase and a component of the PI3K/Akt signaling pathway, were found by Lee et al. in a targeted sequencing experiment (*n* = 240). One sample carried a *PIK3CA*^*E542K*^ mutation (0.4%), 24 p.E545A mutation (10%) and 138 concomitant *BRAF*^*V600E*^ and *PIK3CA*^*E454A*^ mutations (57.7%) [73]. Independently, Wang et al.*,* found 20 samples carrying the *PIK3CA* copy gain mutation (14%, *n* = 141) [74].

#### *RET* proto-oncogene

The *RET* proto-oncogene encodes a tyrosine kinase receptor [75, 76], and *RET* activation promotes downstream signaling, leading to cell proliferation, differentiation and survival. [75]. Depending on the length of the C-terminus of the RET protein, three splice variants of the *RET* mRNA can be distinguished, namely, *RET9*, *RET43* and *RET51*, and all present different cellular localization and function [77]. In PTC, gene fusions are the most common, but *RET* gene mutations were also associated with tumorigenesis, specifically *RET* G691S (rs1799939), L769 L (rs1800861) and S904S (rs1800863) [78]. Khan et al.*,* suggested that rare variants G691SA and S904S are more prevalent in PTC and might be associated with a predisposition to TC development, as opposed to the underrepresented L769 L variant. However, this study was conducted on blood samples of post-thyroidectomy patients, thus the sensitivity of the assay remains to be determined.

### Gene fusions

#### *RET/PTC* gene fusions

The variants of *RET* rearrangements are characterized by the fusion of the kinase domain to the 5′ terminus of the donor gene, resulting in a change of the subcellular localization of the receptor to the cytosol and leading to constitutive activation of the MAPK signaling pathway [79]. Until now, 25 fusion variants were described, 19 of which are associated with PTC [33, 80–92]. The *RET* kinase domain and 5′ end of *CCD6* gene (*RET/PTC1*) fusion [84] or the nuclear receptor co-activator 4 gene (*NCOA4*) (*RET/PTC3*) are most common [81]. Zou et al.*,* reported a 14% rate of *RET/PTC* rearrangement and co-occurrence of *BRAF*^*V600E*^ with *RAS/PTC1* (*n* = 82) [93]. Rossi et al., tested fine-needle aspiration of PTC samples by real-time PCR and showed that in 7.3% of the 940 samples, either *RET/PTC1* or *RET/PTC3* was present [37]. Six of the patients had both *RET* rearrangement and *BRAF* mutation. *RET* rearrangement appears to be fairly common in children with PTC [67]. Out of 13 samples in the study, *RET* gene fusions were detected in 2 (15%) samples by fluorescence in situ hybridization (FISH) assay.

#### KAZN–C1ORF196

Le Pennec et al.*,* identified 4 novel gene fusions, most prominently *KAZN–C1ORF19*6 [94], and this finding was confirmed in both a case study and in 85% of additional PTC samples (*n* = 94). *KAZN* encodes a keratinization-associated adhesion protein, whereas *C1ORF19*6 is a putative gene. The biological function of such gene fusion is unknown, but it is predicted to be a result of an alternative splicing event generates a transcript coding for an in-frame protein. RNA sequencing of 115 samples from thyroid tumor tissues and metastases was performed, and 87 samples classified as PTC were sequenced using the Sanger method to validate the existing mutations [94]. *KAZN–C1ORF19*6 gene fusion was absent in both tumor-adjacent (*n* = 37) and normal thyroid tissue (*n* = 23). Other mutations specific for the patients were identified, all of which highlight the tumor genetic heterogeneity. What is remarkable about this study is the fact that most of the mutations found were specific for a particular patient only.

### Mutations of DNA-repair genes

#### CHEK2

Mutations in DNA repair genes appear to be mutually exclusive with MAPK activator mutations such as *BRAF*^*V600E*^, but they might exist simultaneously with other mutations involved in the MAPK signaling pathway, e.g., *RAS* (see below) [95]. Disruption of DNA repair can be a prognostic marker for aggressive PTC development, according to TCGA (See Table 2) [33]. Genotyping of a Polish population showed that 15.6% of samples (*n* = 468) had one of four cell cycle checkpoint kinase 2 (*CHEK2*) mutations known to contribute to carcinogenesis (truncating mutations IVS2 + 1G > A, 1100delC or del5395 and a mis-sense mutation I157T) [11]. Wójcicka et al.*,* identified the rs17879961 variant as a risk allele for PTC in a group of 1781 patients (OR = 2.2, *P* = 2.37·10^− 10^) [96]. In a Greater Poland female population (case/control: 602/829), the c.470C (I157T) homozygous variant was shown to increase the risk of developing PTC by nearly 13-fold (OR = 12.81, *P* = 1.9·10^− 2^) and was observed in 3 women (0.57%), as determined by pyrosequencing [97]. A heterozygous variant of the same mutation increases the risk by 2-fold (OR = 1.7, *P* = 1.7·10^− 2^).This association was not observed for male patients.

### Alterations in cell signaling pathways

#### RAS

Mutations in the family of RAS proteins are associated with AKT phosphorylation and result in preferential activation of the PI3K/AKT pathway in TC by evasion of apoptosis, proliferation and cellular growth [98, 99]. The RAS family consists of 4 proto-oncogenes: *H-RAS*, *N-RAS*, *K-RAS4A* and *K-RAS4B* [100]. Although *RAS* mutations are more prevalent in FTC, they are also observed in a subset of PTCs [101]. Zou et al.*,* detected *KRAS* mutations (p.Q61R and p.S65 N) in 2 samples (2%, 2/88) and an *NRAS* (p.Q61R) mutation in 3 cases (PTC 1%, TCV 2%). Rossi et al.*,* observed 3.4% of samples harboring a somatic *RAS* mutation (*n* = 940), which correlated with an aggressive histotype and poorer prognosis [37]. Until now, *RAS* mutations have not been found in juvenile thyroid tumors [67].

#### MUC1

Mucin (*MUC1*) plays a role in the signaling pathways of proliferation and differentiation of epithelial cells and is crucial in metastasis and tumorigenesis of epithelial cancers such as adenocarcinomas and ovarian cancer [102]. In PTC, *MUC1* is thought to be a marker of poorer outcome (See Table 2), although this stance is controversial. Using pyrosequencing, Renaud et al.*,* showed that 40% of 94 PTC samples overexpressed *MUC1* in the cytoplasm, which correlated with the presence of the *BRAF*^*V600E*^ mutation in 95% of samples.

### Deregulation of protease expression

#### TMPRSS4

Transmembrane protease serine 4 (TMPRSS4) is a type II transmembrane serine protease overexpressed in several cancer types, including gastric [103], breast [104], lung [105] and thyroid cancers [105–107]. TMPRSS4 promotes cell proliferation, invasion, metastasis and epithelial-mesenchymal transition (EMT) and is predominantly overexpressed in PTC. Kebebew et al.*,* tested 131 tumors by cDNA microarrays, and *TMPRSS4* was one of the 6 genes deregulated in malignant tumors [107]. Jarząb et al.*,* tested 50 samples from 33 patients (23 PTC, 10 other thyroid malignancies) paired with normal tissue using microarray analysis [106]. *TMPRSS4* was classified as one of the genes forming a set of markers that distinguish between benign and malignant tumors.

### Mutations in transcription regulators

#### EIF1AX

Eukaryotic translation initiation factor 1A/X-linked (EIF1AX) is a major player in the transfer of Met-tRNAf and has a high mutation rate in PTC (1.5%, 6/402). *EIF1AX* is suggested as a potential driver of tumorigenesis in other cancers, e.g., uveal melanoma [33, 108, 109], and in TC, it is a promising biomarker candidate. This observation is supported by Karanamurthy et al.*,* who detected *EIF1AX* mutation in 2.3% (*n* = 3/86) of tested PTC samples and 1 of 5 PTC FNA samples using NGS [110]. Almost all of the *EIF1AX* mutations were located at a hotspot A113_splice site at intron 5/exon 6.

#### FOXE1

The thyroid transcription factor forkhead box E1 (FOXE1) possesses a well-conserved DNA binding domain (FDH) and is crucial in the development of a healthy thyroid [111]. Deregulation of transcription factors from the FOX family is recognized as an important element of TC progression.

Penna-Martinez et al., used PCR to genotype 196 PTC samples (German population) for the presence of two known susceptibility SNPs in *FOXE1* [17, 112]. The rs965513 phenotypes “AA” and “AG” were more common in DTC patients in contrast to the “GG” phenotype, which was common in healthy controls. The rs965513 variant is more pronounced in PTC than in FTC [112]. Mond et al.*,* sequenced 120 PTC tumors for SNPs in the coding region of *FOXE1*. Four mis-sense mutations were found in the FHD (c.821C > A, p.P54Q; c.943A > C p.K95Q; c.994C > T, p.L112F), each in a single tumor. Molecular modeling of the described mutations showed their location in a region highly conserved across species, thus explaining the potential carcinogenic effect [111].

### *TERT* promoter

Telomerase reverse transcriptase (TERT) is a catalytic subunit of telomerase vital for the gain of immortality by cancer cells [113, 114]. Two mutations located in the *TERT* promoter region are associated with carcinogenesis, namely, C-to-T substitution (C1,295,228 T) and C-to-A substitution (C1,295,250A) [115]. *TERT* promoter mutations appear to be rare in PTC (4.4%, *n* = 455, Chinese population) [64], but they correlate positively with aggressiveness of the tumor and patient age (See Table 2). These results confirm studies performed by Liu et al.*,* [116, 117]. *TERT* mutations are less common in PTC (11.3%, *n* = 408) than in ATC (42.6%, *n* = 54) when pooled data are considered [118]. Studies also show that *TERT* promoter mutations correlate with poorer outcomes and an increase in aggressiveness of the tumor, even if they do not coincide with *BRAF* mutation [115, 119]. *TERT* promoter mutations are most common in TCV.

### Regulatory RNAs

RNA-mediated regulatory pathways disrupted in carcinogenesis involve micro-RNA (miRNA, miR) signaling. Micro-RNAs are short, 21–23 nt, non-coding endogenous RNA fragments that regulate expression at the posttranscriptional level [120]. MicroRNA-deregulated thyroid cancers are collected in Table 3. T Yoruker et al.*,* used RT-PCR to test serum from pre- and post-operative PTC patients to measure the level of micro-RNA expression [121]. The PTC patient sera levels of 4 miRNAs (miR-222, miR-31, miR-151-5p, let-7) were significantly higher compared with healthy controls, and the miR-21 level was lower (see Table 2). General levels of all miRNAs were lower in the post-operative samples and showed no significant difference with the healthy control group. A similar study was performed by Lee et al.*,* to measure the expression of miR-222 and miR-146b in plasma and tumor tissues [122]. In recurrent tumors, miRNAs were significantly up-regulated compared with non-recurrent patients and healthy controls. Plasma miRNAs levels decreased after thyroidectomy in both cases. The results, especially miR-222 overexpression, confirm the results of other groups [123, 124], suggesting that both miRNAs might be used as biomarkers of cancer progression. MiR-221, miR-22, and miR-21 are involved in *PTEN* regulation [125], whereas miR-126 is associated with angiogenesis [120], and its expression in PTCs as well as undifferentiated thyroid cancers showed a correlation between miR-126 down-regulation and overexpression of VEGF-A mRNA and protein in tumors. miR-639 expression was upregulated in cancer tissues [126]. In contrast, expression of miR-20b a regulator of the MAPK/ERK signaling pathway with potential tumor suppressor qualities, was down-regulated in TC [127]. Samsonov et al.*,* showed the potential differentiating miRNAs (miR-21 and miR-181a) that might be useful in distinguishing PTC from FTC [128]. Studies conducted by Hu et al.*,* associated down-regulation of miR-940, miR-15a, and miR-16 with PTC phenotype [129].

**Table 3:** microRNAs differentially expressed in PTC and their tissue of origin

Up-regulation	Localization	Sample origin	Down-regulation	Localization	Sample origin
let-7	19q13.41	serum	miR-15a	13q14.2	tumor tissue
miR-31	9p21.3		miR-16	13q14.2, 3q25.33	
miR-151-5p	8q24.3		miR-21	17q23.1	serum
miR-146b	10q24.32	plasma, tumor tissue	miR-126	9q34.3	tumor tissue
miR-221	Xp11.3		miR-940	16p13.3	
miR-222	Xp11.3				
miR-639	19p13.12	tumor tissue			

### Follicular thyroid carcinoma (FTC)

Follicular thyroid carcinoma is the second most common thyroid malignancy, is considered more aggressive than PTC, and has a 95% 5-year survival rate. Mortality rate and disease aggressiveness increase with the age of the patient at diagnosis [130].

Hou et al.*,* showed the occurrence of *PTEN* (7%, 6/86 samples) and *PIK3CA* (6%, 5/85 samples) mutations in FTC [42]. *PIK3CA* gene copy gain was found in 20% of tested samples (24/85). These mutations might affect the activation and regulation of the PI3K/Akt pathway. In contrast to PTC, the *BRAF*^*V600E*^ mutation is generally rare in FTC [115]. *TERT* promoter mutations (see Table 4) were also tested, but the FTC sample number was low (20 minimally invasive FTCs without metastasis and 3 FTCs with metastasis). Nevertheless, the results correlated positively with the presence of distant metastases (1/2 minimally invasive samples with distant metastases).

**Table 4:** Somatic mutations found in FTCs. SNV: Single nucleotide variant

Gene	Localization	Gene function	Mutation	Defect in cancer	Clinial correlation	Literature
Gene						
*ARNT*	1q21.3	N/A	CNV	unknown	N/A	Świerniak et al.*,* 2016
*CHEK2*	22q12.1	protein kinase	SNV, (C29,108,001A)	gain of immortality	N/A	Świerniak et al.*,* 2016, Wójcicka et al.*,* 2014
*COL1A1*	17q21.33	pro-alpha1 chain of type I collagen	indel, chr17: 48275120	unknown	N/A	Świerniak et al.*,* 2016
*COX6/DERL2*	*COX6/A1:* 12q24.31, *COX6/A2:* 16p11.2, *DERL2*: 17p13.2	N/A	translocation	unknown	N/A	Świerniak et al.*,* 2016
*FBXW7*	4q31.3	subunit of ubiquitin protein ligase complex called SCFs	CNV	unknown	N/A	Świerniak et al.*,* 2016
*FOXO4*	Xq13.1	suppressor of transcription	SNV, (C70,321,204 T)	Deregulation of transcription, alters protein structure	N/A	Świerniak et al.*,* 2016
*IDH1*	2q34	catalyzes the oxidative decarboxylation of isocitrate to 2-oxoglutarate	LOH	unknown	N/A	Świerniak et al.*,* 2016
*JAK3*	19p13.11	Protein kinase	intronic region	unknown	N/A	Świerniak et al.*,* 2016
*KAZN-C1ORF196*	*KAZN*: 1p36.21, *C1ORF196*: 1p36.21	unknown	Gene fusion	unknown	N/A	Salajegheh et al.*,* 2016
*KTN1*	14q22.3	membrane protein involved in organelle motility	deletion in chr14:56139994	unknown	N/A	Świerniak et al.*,* 2016
*MITF*	3p13	transcription regulator	insertion, chr3:69987750	unknown	N/A	Świerniak et al.*,* 2016
*NCOA2*	8q13.3	epigenetic modifier	chr8 position 71,053,835 A > C	unknown	N/A	Świerniak et al.*,* 2016
*PAX8/PPARG*	*PAX8: 2q14.1, PPARG: 3p25.2*	N/A	t(2;3)(q13;p25) translocation	competitor inhibitor of PPARγ/ transcription factor similar to endogenous PPARγ	N/A	Lacroix et al.*, 2*005, Giordano et al.*,* 2006
*PIK3CA*	*3q26.32*	catalytic subunit of phosphatidylinositol 3-kinase	CNV (gain)	unknown	N/A	Hou et al.*,* 2007
*TMPRSS4*	11q23.3	serine protease	overexpression	Promotes cancer cells proliferation, invasion and metastasis	positive correlation with staging of tumor nodes metastasis	Guan et al.*,2*015
*TERT* promoter	5p15.33	telomerase reverse transcriptase	C228T (rs35809415), C250A, C250T(rs1020948523)	unknown	presence of metastases	Bae et al.*,* 2016
*USP6*	17p13.2	ubiquitin Specific Peptidase	CNV	unknown	N/A	Świerniak et al.*,* 2016
*WRN*	8p12	repair od double stranded breaks	LOH	unknown	N/A	Świerniak et al.*,* 2016
Regulation of expression						
miR-199a-5p	19p13.2	regulator of *CTFG* in healthy cells	Micro RNA	Disruption of regulatory pathways, propagation of cancer	Downregulation during tumorigenesis	Sun et al.*,* 2016

Świerniak et al.*,* performed targeted NGS sequencing of 48 FTC tumors [12]. The authors identified previously undescribed somatic mutations in both intronic and exonic regions. FTC mutations were found in *FOXO4* (transcription suppressor), *CHEK2* and *NCOA2* (epigenetic modifier) genes. Additionally, 10/18 identified single nucleotide variants (SNVs) were located in the non-coding regions of the studied genes. Other types of mutations included indels in *MITF* and *KTN1* genes (transcription factor and transmembrane kinesin receptor, respectively) and loss of heterozygosity (LOH) in the *IDH1* gene that belongs to the dehydrogenase family. Copy number variations (CNV) in *ARNT* (facilitates transport to the nucleus, transcriptional co-regulator of *HIF1* expression), *FBXW7* (component of the ubiquitin degradation signaling chain) and *USP6* (ubiquitin specific peptidase) were also found in samples with populations of cells highly represented in tumors. In the low-confidence FTC group, a distinct subset of mutations was found, meaning that the differentiation of the two subsets based on their molecular profiles might be possible. In lower-confidence FTC, subset mutations were found in the *COL1A1* gene, which is a fibrin-forming type of collagen. LOHs were identified in *WRN* (belonging to a family of DNA and RNA helicases) and *PPARγ* (member of a nuclear receptor subfamily), among others. A new translocation of unknown function was described, namely, *COX6C/DERL2*. *KAZN/C1ORF196* gene fusion was confirmed in the case study and in 55% (out of 11) of FTC additional samples [94].

One of the most common genetic events in follicular thyroid cancer is the gene fusion of *PAX8/PPARγ* or PPFP oncoprotein gene [131, 132]. PAX8 on its own is necessary for the normal development of the thyroid [133], and PPARγ is a nuclear receptor [134]. PAX8/PPARγ fusion is present in 35% of FTC tumors on average, can be overexpressed by up to 50-fold compared with endogenous *PPARγ* in tumor tissues [135, 136] and is probably the effector component of the oncogenic rearrangement [137].

In FTC, as in PTC, overexpression of *TMPRSS4* is observed in 53.6% (15/28) of the samples, as shown by Guan et al. [138].

Sun et al.*,* found a positive correlation between FTC tumorigenesis and low levels of miR-199a-5p expression [131]. MiR-199a-5p was identified as a regulator of the connective tissue growth factor (*CTFG*), which acts as an inhibitor of the cell cycle in healthy tissue. In tumor conditions, both fusion proteins appear to possess binding domains that retain their function in the correct cellular context [132].

### Anaplastic thyroid carcinoma (ATC)

Anaplastic thyroid carcinoma is the most aggressive type of TC and contributes to 1–2% of all thyroid cancers and 39% of reported deaths [133]. The 6- to 12-month mortality rates reach 80%. The high aggressiveness of ATC is caused by dedifferentiation of well-differentiated thyroid cancer forms such as PTC [134–136]. Compared with PTC and poorly differentiated thyroid cancers, the mutation burden in ATC is much larger [137] (see Table 5).

**Table 5:** Somatic mutations found in ATCs

Gene	Localization	Gene function	Mutation	Defect in cancer	Physiological effect	Literature
Chromosomal Abberation						
*KAZN*-*CIORF196*	1p36.21, 1p36.21	N/A	Gene fusion	potential role in progression and development of tumors		Le Pennec et al.*,* 2015
Gene						
*ARID1A, ARID1B, ARD2, ARID5B, SMARCB1, PBRM1, ATRX*	1p36.11, 6q25.3, N/A, N/A, 22q11.23, 3p21.1, Xq21.1	components of the SWI/SNF complex, responsible for the chromatin remodeling	N/A	mutation in one of the complex components leads to dysfunction of the whole complex	N/A	Landa et al.*,* 2016, Latteyer et al.*,* 2016
*ALK*	2p23.1	anaplastic lymphoma kinase	D1203H	hallmark of anaplastic tumors	N/A	Bonhomme et al.*,* 2017, Latteyer et al.*,* 2016
*ATM*	11q22.3	cell-cycle checkpoint, response to DNA damage	E2039K	higher mutation burden, consistent with the lack of checkpoint function	N/A	Landa et al.*,*2016, Kunstman et al.*,*2015
*BRAF rs113488022*	7q34	serine/threonine kinase, response to cell growth factors	V600E	constitutive activation of MAPK pathway	N/A	Santarpia et al.*,* 2008, Guerra et al.*,* 2013, Kasaian et al.*,* 2015, Landa et al.*,* 2016, Latteyer et al.*,* 2016
*DAXX*	6p21.32	transcription repressor binding the sumoylated transcription factors	S641X	potential driver mutation	correlates with non-thyroidal malignancies	Kunstman et al.*,* 2015
*EIF1AX*	Xp22.12	translation initiation factor, transfer of met-trnaf	Splice site 1 bp upstream of ex6 (C > G), G9R (C > G), P2R(G > C)	potential driver mutation	N/A	Kunstman et al.*,* 2015, Landa et al.*,* 2016
*ERBB2*	17q12	downstream enhancer of kinase-mediated signaling pathways	D387N	potential driver mutation	N/A	Kunstman et al.*,* 2015
			D873N, A763T		N/A	Bonhomme et al.*,* 2017
*HECTD1 rs769574276*	14q12	ubiquitin protein ligase	L547 V	impairment of ubiquitynylated proteins degradation	N/A	Kunstman et al.*,* 2015
*KMT2A, KMT2C, KMT2D (MLL2), SETD2*	11q23.3, 7q36.1, 12q13.12, 3p21.31	histone methyltransferases, epigenetic modifiers	N/A, KMT2D: Q1892Q (rs753626919), R5389W	impairment of epigenetic mechanisms, potential driver mutation	N/A, KMT2D: correlates with non-thyroidal malignancies	Landa et al.*,* 2016, Kunstman et al.*,* 2015
*MET*	7q31.2	tyrosine-protein kinase met	I166T	proto-oncogene	N/A	Bonhomme et al.*,* 2017
*mTOR*	1p36.22	response element = to stress, possessing kinase activity	R164Q (rs573705289), M2327I	potential driver mutation	correlates with non-thyroidal malignancies	Kunstman et al.*,* 2015
*NF1*	17q11.2	neurofibromatosis related gene	P2696L (rs778799019), R2496X (rs752162999)	potential driver mutation	correlates with non-thyroidal malignancies	Kunstman et al.*,* 2015, Landa et al.*,* 2016, Latteyer et al.*,* 2016
*NOTCH1–4* (*NOTCH2* in Kunstman)	1p12	transmembrane receptors	NOTCH2: S361F (rs587735797), R1393H	potential driver mutation	correlates with non-thyroidal malignancies	Kunstman et al.*,* 2015, Landa et al.*,* 2016
*PIK3CA*	3q26.32	PI3K/AKT/mTOR pathway effector	E542K (rs121913273), E545K (rs104886003)	mutation of helical domain	N/A	Landa et al.*,* 2016, Kunstman et al.*,* 2015, Hou et al.*,* 2007
*PTEN*	10q23.31	PI3K/AKT/mTOR pathway effector	N/A	truncated protein	N/A	Landa et al.*,* 2016, Hou et al.*,* 2007
*RAS*	11p15.5, 1p13.2, 12p12.1	signal transduction	N/A	preferential activation of PI3K-AKT pathway	N/A	Santarpia et al.*,* 2008, Guerra et al.*,* 2013, Landa et al.*,* 2016, Latteyer et al.*,* 2016, Hou et al.*,* 2007
*TERT* promoter	5p15.33	telomerase reverse transcriptase	C228T (rs35809415), C250T (rs1020948523)	gain of immortality	shorter survival	Bae et al.*,* 2016, Landa et al.*,* 2016
*TMPRSS4*	11q23.3	serine protease	N/A	promotes proliferation	positive correlation with tumor grade	Guan et al.*,* 2015
*TP53*	17p13.1	tumor suppressor protein	Y163C (rs148924904)	gain of immortality	N/A	Kasaian et al.*,* 2015, Landa et al.*,* 2016, Bonhomme et al.*,* 2017
*USH2A*	1q41	uscherin, extracellular matrix binding protein interacting with collagen and fibronectin	I2189V (rs542406401), D798V (rs148431156), E571K(C > T), L1727F(G > A)	missense mutations	N/A	Kunstman et al.*,* 2015
*CREBBP*	16p13.3	histone acetyltransferase	N/A	epigenetic modifier	N/A	Landa et al.*,* 2016
*EP300, BCOR, BCL6*	22q13.2, Xp11.4, 3q27.3	epigenetic modifiers	N/A	abnormal protein modifications	N/A	Landa et al.*,* 2016
*CTNNB1*	3p22.1	cytoskeletal anchor, adhesive junctions	Q108H	unknown	N/A	Kunstman et al.*,* 2015, Landa et al.*,* 2016
*MSH2*	2p21	DNA mismatch repair	N/A	gain of mutation phenotype	N/A	Landa et al.*,* 2016, Kunstman et al.*,* 2015
*MSH5*	6p21.33		A199V (C > T)		N/A	
*MSH6*	2p16.3		D736H (G > C)		N/A	
*MLH1*	3p22.2		I19M (C > G), I68M (rs780141938), Q60X (C > T)		N/A	
*MLH3*	14q24.3		L264 V (G > C)		N/A	

ATC can arise independently, but it often coincides with well-differentiated tumors. Co-occurrence of *BRAF* and *RAS* mutations in ATC suggests its common genetic origin with DTC [135, 139, 140]. Hou et al.*,* tested 50 ATC tumors and found a high prevalence of mutations associated with PI3K/Akt pathway activation: *PTEN* 16% (8/50) and *PIK3CA* 12% (6/50) [42]. *RAS* mutations were also identified in 8% (4/50) of samples. The molecular heterogeneity of ATC makes it incredibly difficult to analyze. Kasaian et al.*,* performed whole-genome sequencing of 1 ATC sample and identified 24 somatic mutations, including two heterozygous mutations in *BRAF* (V600E) and *TP53* (Y163C) genes. [141]. Kunstman et al.*,* tested 22 tumor samples with whole-exome sequencing [142]. The majority (68%) of the observed variants code for mis-sense mutations. A total of 16 genes were identified as potential drivers of tumorigenesis, 6 of which were present in multiple samples, namely, *NF1* (negative regulator of RAS pathway), *mTOR* (kinase, mediates response to stress), *ERBB2* (EGF receptor), *DAXX* (apoptosis regulator and transcription repressor among other functions), *MLL2* (histone methyltransferase), and *NOTCH2* (regulator of cell fate). In addition, recurrent mutations of *EIF1AX* and *HECTD1* (ubiquitin-transferase activity) and non-synonymous *USH2A* (development of retina and inner ear) mutations were observed. Several of the tested cases presented a hypermutation phenotype, resulting in a high mutation burden of mismatch repair genes. Bonhomme et al.*,* sequenced 94 ATC tumors targeted to *TERT* using NGS and 98 samples using Sanger sequencing [143]. More than 50% of samples possessed the *TP53* mutations, and *ALK* rearrangements were rare. In total, 210 different alterations were found, including those not previously described in the context of TC, such as *MET* (proto-oncogene) and *ERBB2* mutations. In the Korean population, 60% samples (3/5) had a *TERT* promoter mutation, which coincided with BRAF^V600E^ [115]. In a study by Landa et al.*,* the presence of *BRAF*^*V600E*^ mutation was observed in 45% out of 33 tumors [137]. In the same study *RAS* mutations (*H-RAS*, *K-RAS,* and *N-RAS*) occurred in 24% of the samples but were mutually exclusive with *BRAF*^*V600E*^.

Other mutations found in ATCs were *NF1* (3 samples), *PIK3CA* (18%), and *PTEN* (15%). *PIK3CA* mutation tends to co-occur with *BRAF* mutations, whereas *NF1* tends to be present simultaneously with *PTEN* mutations. *EIF1AX* mutations were present in 9% of the 33 studied tumors.

For the first time, Landa et al. reported mutations in components of the SWI/SNF complex (chromatin remodeling system), as reported in 36% (*n* = 33) of tumors. Mutations were also found in histone methyltransferase genes (*KMT2A*, *KMT2C*, *KMT2D*, and *SETD2*) in 24% (n = 33) of ATCs. Additional genes involved in epigenetic processes, i.e., *CREBBP, EP300, BCOR,* and *BCL6,* were mutated at low frequencies*.* One sample carried a *CTNNB1* (p.L347P; WNT signaling pathway) mutation, but this finding was not validated by others. Mutations were also observed in members of the MMR DNA repair pathway (*MSH2*, *MSH6*, and *MLH1*) in 12% of samples. Another DNA damage response element, *ATM,* was mutated in 9% of tested ATCs. Landa et al.*,* reported frequent (73%, n = 33) *TERT* promoter and *TP53* mutations. The *TERT* promoter C228T variant was more common than the C250T variant. *TERT* promoter mutations significantly diminished the survival rate from 732 to 147 days.

Gene fusions are also present in ATC. *KAZN/C1ORF196* was identified by Le Pennec et al.*,* in a case study and confirmed in 11% of additional ATC samples [94]. Guan et al.*,* observed an increase of *TMPRSS4* expression in all ATC samples (*n* = 12) compared with adjacent normal tissue [138]. Targeted DNA sequencing for *TP53*, *RAS*, *BRAF*, *ALK,* and *NF1* of 30 formalin-fixed paraffin-embedded (FFPE) ATC tumor samples by Latteyer et al.*,* showed that 28/30 tested samples carried at least one of the tested mutations [144]. *TP53* mutation was most common (18/30), followed by *NF1* (11/30) and *RAS* family mutations (7/30 combined). It is also worth mentioning that nearly a third of the samples showed residual contaminations of either PTC or FTC tissue, proving the anaplastic tumor heterogeneity.

Zhang et al.*,* tested the expression of myocardin family genes (involved in cell growth arrest, inhibition of differentiation, metastasis and tumor invasion) [145]. *MRTF-A* was overexpressed in metastatic ATC but was not present in either in primary tumor or the adjacent tissue. Following this finding, down-regulation of miR-206 was identified as the factor leading to the *MRTF-A* overexpression.

## Conclusions

**Fig. 2:**
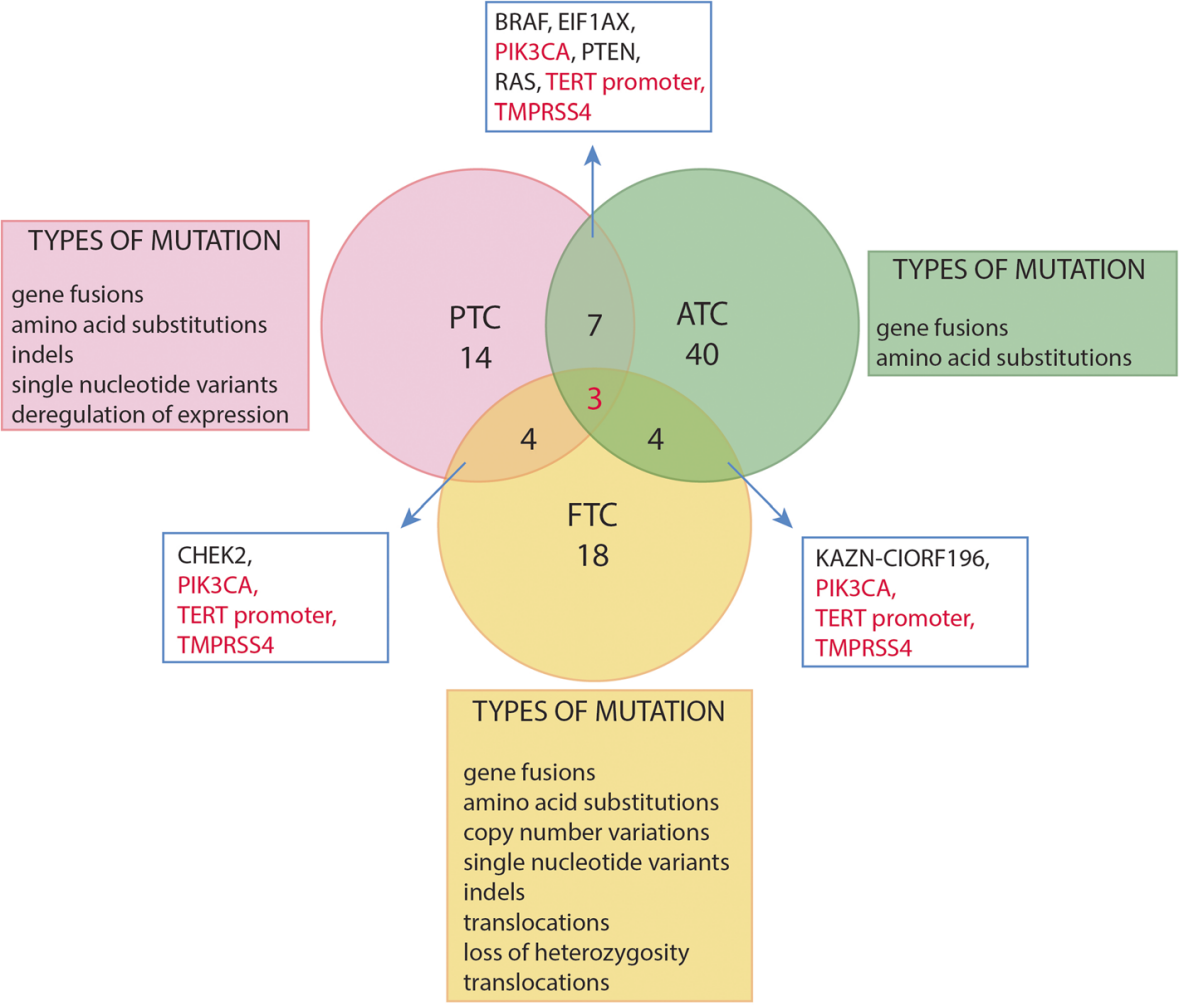
Genetic changes identified in thyroid cancers of follicular origin. Genes common for all three TC subtypes are marked in red

Despite the large number of mutations involved in the tumorigenesis of thyroid carcinomas (Fig. 2), many tumors remain unclassified by FNA biopsy or even genetic testing. Pagan et al.*,* notes that over 50% of samples tested for a large number of reported mutations already observed in TC by RNA-seq do not show a phenotype, leading to the conclusion that the fast-growing database of somatic and driver mutations in thyroid cancers must be expanded with respect to histological subtype [146].

DNA methylation in thyroid cancer has been extensively studied and reviewed but was not discussed in detail in this review. However, it is worth mentioning that the advances in next-generation sequencing and microarray techniques enable in-depth research on the methylation pattern in GC-rich regions and its effect on gene expression. Most studies focus on pre-determined loci [147, 148], and fewer are available at the whole-genome scale [149, 150]. Determination of the methylation patterns can be potentially useful for differentiating between TC subtypes with greater precision. The largest study to date that examines whole-genome methylation was performed as a component of the TCGA project (PTC, *n* = 496) [33]. In a recent study, Bisarro dos Reis et al.*,* proposed a hyper/hypomethylation genetic signature that allows distinction between TC subtypes (Hürtle cell, PTC, FTC, non-neoplastic tissue and benign lesions, ATC) based on the Illumina 45 k platform, with high sensitivity and specificity (63 and 92%, respectively) [151]. Methylation can also be used as a prognostic marker of disease outcome, as proposed in the same article. Beltrami et al.*,* proposed the PTC hypomethylation signature of 41 PTC-paired samples (88% of hypomethylation) as a prognostic biomarker of PTC development [152]. This signature coincides with the presence of the *BRAF*^*V600E*^ mutation (68% of the hypomethylation signature).

In the era of advanced molecular analysis, genetic markers have become a useful tool for the evaluation of thyroid tumor growth and progression. Molecular biomarkers can be applied in the classification of thyroid tumor subtypes and the prediction of disease outcome and might also aid development of systemic molecular therapies in cancers that are refractory to standard treatment. The discovery of specific genetic alterations and mechanisms of thyroid carcinoma development is expected to lead to more personalized treatment for patients with advanced and recurrent disease. Despite the presence of the molecular changes described in this review, the roles of molecular biomarkers in the development of different thyroid tumor subtypes still remain unclear.

## Abbreviations

ATC: Anaplastic thyroid cancer
CNV: Copy number variations
DTC: Differentiated thyroid cancer
FISH: Fluorescence in situ hybridization
FNAB: Fine-needle aspiration biopsy
FTC: Follicular thyroid cancer
GWAS: Genome-wide association study
HRM: High resolution melting
LOH: Loss of heterozygosity
MTC: Medullary thyroid cancer
NGS: Next-generation sequencing
PCR: Polymerase chain reaction
PTC: Papillary thyroid cancer
SNP: Single nucleotide polymorphism
SNV: Single nucleotide variant
TC: Thyroid cancer
TCGA: The Cancer Genome Atlas
TCV: Tall cell variant
